# Insulin-like growth factor family and its impact on pulmonary arterial hypertension: a review

**DOI:** 10.3389/fphys.2025.1679278

**Published:** 2025-10-15

**Authors:** Nuo Li, Degang Mo, Hongyan Dai

**Affiliations:** ^1^ School of Clinical Medicine, Shandong Second Medical University, Weifang, China; ^2^ Qingdao Hospital, University of Health and Rehabilitation Sciences, Qingdao, China; ^3^ School of Medicine, Qingdao University, Qingdao, China

**Keywords:** pulmonary arterial hypertension, insulin-like growth factor, insulin-like GrowthFactor binding protein, insulin-like growth factor binding protein-like, pathogenicmechanisms

## Abstract

Pulmonary arterial hypertension (PAH) is a progressive and life-threatening cardiopulmonary disorder with rising global prevalence and limited curative options. Although current therapies have improved clinical outcomes, they primarily offer symptomatic relief, underscoring the need for novel disease-modifying strategies. PAH pathogenesis involves multiple interrelated mechanisms, including genetic predisposition, endothelial dysfunction, inflammation, oxidative stress, and vascular remodeling. Among these, endothelial dysfunction and vascular remodeling are central to both disease initiation and progression. Endothelial dysfunction is an early and central event, leading to an imbalance between vasodilators and vasoconstrictors, increased vascular permeability, and a pro-thrombotic state. These changes initiate a cascade of vascular remodeling, characterized by pulmonary artery smooth muscle cell proliferation, fibroblast activation, and extracellular matrix deposition, ultimately resulting in increased vascular resistance and right ventricular failure. Recent evidence indicates that the insulin-like growth factor (IGF) family significantly contributes to both vascular remodeling and endothelial dysfunction in PAH. Through complex signaling networks involving IGF receptors and regulatory proteins, IGFs promote smooth muscle cell proliferation, extracellular matrix accumulation, and endothelial cell dysfunction—aggravating vascular alterations characteristic of the disease. While the IGF family—including IGFs, IGFBPs, and IGFBPLs—has been implicated in a range of cardiovascular disorders, its specific involvement in PAH remains insufficiently characterized. This review consolidates current evidence on the IGF family’s roles in PAH pathogenesis, with emphasis on its contributions to vascular remodeling, endothelial dysfunction, and right ventricular adaptation. By delineating the distinct yet interconnected actions of IGF-related molecules, this review aims to identify potential diagnostic biomarkers and therapeutic targets, ultimately advancing precision strategies for PAH management.

## 1 Introduction

Pulmonary hypertension (PH) is a multifactorial and life-threatening cardiopulmonary syndrome characterized by progressive remodeling of the pulmonary vasculature and sustained elevation of pulmonary vascular resistance. In the absence of timely intervention, PH inevitably progresses to right ventricular failure and premature death (Galie et al., 2016; Hassoun, 2021). Current epidemiological evidence indicates that PH affects approximately 1% of the global population. Its prevalence is notably higher among older adults (over 65 years) and populations in developing countries (Hoeper et al., 2016). Given its increasing incidence and poor prognosis, a deeper understanding of the molecular and pathophysiological mechanisms underlying PH is essential to improve disease management and patient outcomes.

PH can be categorized into five distinct groups based on its pathophysiology, clinical presentation, and treatment approaches: 1) pulmonary arterial hypertension, 2) pulmonary hypertension resulting from left-sided heart disease, 3) pulmonary hypertension associated with lung disease or hypoxia, 4) chronic thromboembolic pulmonary hypertension, and 5) pulmonary hypertension with undefined or multifactorial causes (Kovacs et al., 2024; Simonneau et al., 2019). PAH is a condition of particular interest due to the complexity of its pathogenesis, which involves vascular remodeling, smooth muscle cell proliferation, and endothelial dysfunction. PAH is often associated with high morbidity and poor prognosis. Although the exact mechanisms underlying PAH remain incompletely understood, recent studies have identified the IGF family as critical contributors to the disease’s progression. The IGF family, comprising IGF-1, IGF-2, and their respective receptors (IGF-1R and IGF-2R), along with several related binding proteins, plays a central role in regulating cellular processes such as proliferation, differentiation, survival, and apoptosis.

This review has two principal objectives. First, it aims to provide a comprehensive overview of the IGF family and its role in the pathogenesis of PAH. Second, it synthesizes emerging evidence on the molecular mechanisms through which IGF signaling contributes to pulmonary vascular remodeling. By integrating these findings, this review offers mechanistic insights into how IGF ligands, receptors, and binding proteins influence vascular pathology in PAH. Moreover, it highlights the translational potential of the IGF axis as a source of novel therapeutic targets and prognostic biomarkers, thereby contributing to improved patient stratification and clinical outcomes.

## 2 Insulin-like growth factor

### 2.1 Insulin-like growth factor 1(IGF-1)

IGF-1 is a protein that plays a crucial role in growth and development, particularly in the growth of tissues, cells, and organs (Junnila et al., 2013; Lin J. et al., 2023). Moreover, IGF-1 also plays a role in cardiovascular disease. IGF-1 exerts beneficial effects on cardiovascular health through mechanisms such as enhancing endothelial function, attenuating vascular inflammation, and promoting angiogenesis, thereby contributing to the prevention of atherosclerotic progression, restenosis following angioplasty, and ischaemic heart disease (Delafontaine, 1995; Higashi et al., 2010; Colao, 2008).

Numerous studies present divergent views on the role of IGF-1 in PAH. Some research suggests that IGF-1 is involved in the pathophysiology of PAH, with its dysregulated signaling contributing to pulmonary vascular remodeling, smooth muscle cell proliferation, and fibrosis, ultimately exacerbating the disease (Cao et al., 2000; Sun et al., 2016; Bouzi et al., 2021). The study by Martin Connolly and colleagues found that in the MCT-induced PAH rat model, IGF-1 expression in the right ventricle significantly increased. It binds to its receptor and activates downstream signaling pathways such as Akt and mTOR, thereby enhancing protein synthesis, inhibiting protein degradation, and promoting cardiomyocyte hypertrophy, which drives the development of right ventricular hypertrophy. Ultimately, this may lead to right heart failure and exacerbate the progression of PAH. Furthermore, the elevated expression of IGF-1 is closely associated with a decrease in miR-322–5p expression. The suppression of miR-322–5p lifts its inhibitory effect on IGF-1, resulting in increased IGF-1 expression, which further promotes the pathological processes associated with PAH (Connolly et al., 2018). Dewachter et al. reported IGF-1 promotes the proliferation of pulmonary arterial smooth muscle cells (PASMCs) and exacerbates pulmonary vascular remodeling by activating signaling pathways such as phosphoinositide 3-kinase (PI3K)/protein kinase B (PKB/Akt) and mitogen-activated protein kinase (MAPK), regulating cyclin expression, and interacting with other factors (Dewachter et al., 2006). These mechanisms contribute to sustained elevation of pulmonary artery pressure, driving the progression of PAH.

However, a study by Jin et al. found that IGF-1 plays multiple roles in slowing disease progression. Upon binding to its receptor, IGF-1 activates the p38 MAPK pathway, inducing iNOS expression. The nitric oxide (NO) produced by iNOS inhibits apoptosis through mechanisms such as regulating redox status, activating anti-apoptotic proteins, and stabilizing intracellular calcium levels. Additionally, IGF-1 interacts with factors like platelet-derived growth factor (PDGF) to promote the survival and proliferation of PASMCs, reducing the abnormal pulmonary vascular remodeling caused by cell death and survival imbalance, thereby delaying PAH progression (Jin et al., 2014).

Thus, IGF-1 exerts a dual role in PAH. This apparent dichotomy reflects the cell- and context-dependent nature of IGF-1 signaling, influenced by cell type, ligand exposure, and interactions with other factors such as PDGF, TGF-β, and hypoxia. Specifically, in endothelial cells, physiologic IGF-1 concentrations preferentially engage PI3K–Akt–endothelial nitric oxide synthase (eNOS) signaling, enhancing NO bioavailability and promoting endothelial survival (Muniyappa and Sowers, 2012). In contrast, PASMCs and fibroblasts exposed to sustained IGF-1 signaling activate PI3K–Akt–mTOR and MAPK/extracellular signal-regulated kinase (ERK) cascades, driving proliferation and extracellular matrix deposition (Chetty et al., 2006). These effects can be further modulated by regulatory miRNAs such as miR-322–5p and by the stage of disease (Connolly et al., 2018). These modulatory effects are particularly relevant when considering disease progression. For example, transient, low-level increases in IGF-1 signaling in endothelial cells may augment eNOS activity and preserve vasodilator function early in disease, whereas sustained or cell-type-specific overactivation drives proliferative, anti-apoptotic programs later in disease progression (Jin et al., 2014; Higashi et al., 2019). Variations in experimental models, including species, PAH induction methods, and *in vitro* versus *in vivo* conditions, may also contribute to the differing observations across studies. Collectively, these findings emphasize that IGF-1’s role in PAH is highly context-dependent, being protective under certain conditions but potentially pathogenic under others.

### 2.2 Insulin-like growth factor 2(IGF-2)

IGF-2 is a growth factor similar to insulin, primarily active during embryonic development, and involved in cell proliferation and repair in certain adult tissues. It exerts its effects through binding to the IGF-2 receptor (IGF-2R), influencing cellular growth, differentiation, and metabolism (DeChiara et al., 1990; Bergman et al., 2013),and it plays a crucial role in cardiovascular health by promoting cardiac cell growth and repair, influencing vascular smooth muscle cell function, and contributing to heart remodeling, fibrosis, and potential tumorigenesis in certain conditions.

While its role in PAH remains unclear, IGF-2 has been implicated in vascular remodeling and right ventricular hypertrophy in other cardiovascular diseases. Studies have suggested that IGF-2 signaling may contribute to endothelial dysfunction and smooth muscle proliferation, both of which are critical pathological features of PAH (Chisalita et al., 2009; Jang et al., 2022; Tan et al., 2021). However, direct evidence linking IGF-2 to pulmonary arterial hypertension remains limited. A focused search of the recent literature (2023–2025) did not identify studies reporting consistent IGF-2 expression changes in standard preclinical models such as monocrotaline and Su5416+ hypoxia, underscoring the current evidence gap. This gap highlights the need to evaluate its potential role based on established biology. IGF-2 is a putative contributor to pulmonary vascular disease: it shares structural homology and receptor overlap with IGF-1 (binding IGF-1R and IR-A),suggesting it could engage similar signaling networks (Garrett et al., 2019).

We therefore propose the following testable hypothesis: IGF-2 contributes to pulmonary vascular remodeling and right-ventricular adaptation in PAH via IGF-1R/IR-mediated activation of PI3K/Akt and MAPK pathways, in a cell-type- and time-dependent manner (Blyth et al., 2020; Liu et al., 2001). To validate this hypothesis, future studies should (i) quantify IGF-2 expression and localization in patient lungs and across time points in preclinical models; (ii) apply genetic and pharmacologic loss-of-function approaches (e.g., IGF-2 knockout or neutralizing antibodies) to define its causal role; and (iii) perform cell-type–specific mechanistic studies (endothelial cells, PASMCs, adventitial fibroblasts) combining pathway readouts with phenotypic assays of proliferation, apoptosis, and extracellular matrix remodeling. These efforts would clarify the role of IGF-2 in PAH pathogenesis and inform its potential as a therapeutic target.

Table 1 summarizes the IGFs associated with the PAH.

**TABLE 1:** The role that IGFs play in biological functions, cardiovascular functions and PAH.

Biomarkers	References	Biological functions	Cardiovascular functions	Role in PAH
IGF-1	Junnila et al. (2013) Higashi et al. (2010)	IGF-1 plays a crucial role in promoting growth development, and tissue repair	IGF-1 contributes to the prevention of atherosclerotic progression restenosis, follows angioplasty and ischaemic heart disease	Its dysregulated signaling exacerbates the PAH process. But it can also delay disease progression by inhibiting apoptosis and alleviating vascular remodeling
IGF-2	DeChiara et al. (1990) Chisalita et al. (2009)	IGF-2, similar to insulin, exerts its effects through binding to the IGF-2R, influencing cellular growth, differentiation, and metabolism	IGF-2 promotes cardiac cell growth and repair, influencing vascular smooth muscle cell function, and contributing to heart remodeling, fibrosis, and potential tumorigenesis	Its role in PAH remains unclear^a^

^a^ PAH, pulmonary arterial hypertension; IGF-1, insulin-like growth factor 1; IGF-2, insulin-like growth factor 1; IGF-2R, insulin-like growth factor 2 receptor.

## 3 Insulin-like growth factor binding proteins (IGFBPs)

### 3.1 Insulin-like growth factor binding protein 1(IGFBP-1)

IGFBP-1 is known to regulate the bioavailability and activity of IGFs, including IGF-1 and IGF-2 (Lewitt and Boyd, 2024). Although direct studies on IGFBP-1 in PAH are limited, its role in modulating IGF signaling suggests that it may contribute to the pathophysiology of PAH. By binding to IGFs, IGFBP-1 may influence smooth muscle cell proliferation and migration, processes that are central to the vascular remodeling observed in PAH (Slater et al., 2019). Additionally, given its involvement in metabolic regulation and insulin resistance, elevated levels of IGFBP-1 may further exacerbate the pathological changes seen in PAH, but this hypothesis requires further investigation.

### 3.2 Insulin-like growth factor binding protein 2(IGFBP-2)

IGFBP-2 is a member of the IGF family. It is a critical modulator of the IGF axis, primarily through its high-affinity binding to IGF-1 and IGF-2, thereby regulating their bioavailability and interaction with IGF receptors. In addition to its IGF-dependent functions, IGFBP-2 exerts IGF-independent functions via interactions with integrins and activation of downstream signaling pathways such as PI3K/Akt. It plays a significant role in regulating cell proliferation, metabolism, and vascular remodeling. Notably, IGFBP-2 exhibits context-dependent biological effects: it may promote tumor progression and pathological vascular changes in cancer and cardiovascular disease, while exerting protective metabolic effects in conditions such as insulin resistance and obesity. This dual functionality underscores its complex and multifaceted role in human physiology and disease pathogenesis (Byun et al., 2016; Chin et al., 2023; Li et al., 2020; Guiot et al., 2016).

Many studies have shown a strong correlation between IGFBP-2 levels and disease progression in PAH (Nies et al., 2022; Griffiths et al., 2020). Yang et al. demonstrated that patients with PAH had significantly elevated serum levels of IGFBP-2, a change closely associated with disease severity and patient survival prognosis. IGFBP-2 expression was also significantly increased in lung tissues and secreted by PASMC. IGFBP-2 may contribute to the pathogenesis of PAH through several mechanisms, including the stimulation of cellular growth via non-IGF-dependent pathways, its association with inflammation-related cardiorespiratory disorders, and its regulation of phosphatase and tensin homolog (PTEN) in vascular smooth muscle cells (Yang et al., 2020). Also, Beate Christiane Schlueter’s article revealed that levels of the protein known as IGFBP-2 were elevated in patients diagnosed with PAH in comparison to individuals deemed to be healthy. Furthermore, the extent of this elevation in IGFBP-2 levels appeared to be associated with the severity of the disease (Schlueter et al., 2024).

In conclusion, IGFBP-2, with significantly elevated serum levels in PAH, may play a role in the pathogenesis of the disease through multiple pathways. Its correlation with disease severity and survival makes it a potential biomarker and therapeutic target.

### 3.3 Insulin-like growth factor binding protein 3(IGFBP-3)

IGFBP-3 is the most abundant member of the IGFBP family, primarily functioning to regulate IGF bioavailability through direct binding while also modulating cell proliferation and apoptosis via IGF-independent mechanisms. Additionally, IGFBP-3 plays a crucial role in tumor suppression, with its reduced expression being closely associated with the onset and progression of various malignancies. It is also essential for bone growth and metabolic regulation (Deng et al., 2018). Further studies have highlighted its significant involvement in the cardiovascular system, where it contributes to vascular remodeling, endothelial function modulation, and the progression of atherosclerosis (Ding et al., 2023).

Furthermore, IGFBP-3 has garnered attention for its regulatory role in various cardiovascular conditions, particularly in influencing vascular remodeling, smooth muscle cell proliferation, and endothelial function (Rhee et al., 2021). However, while the alteration of IGFBP-3 expression has been associated with atherosclerosis and other vascular diseases, its involvement in PAH remains poorly understood. Given the crucial role of IGFBP-3 in regulating IGF bioavailability and modulating cellular responses via IGF-independent mechanisms, it is plausible that IGFBP-3 may impact the development of PAH through similar pathways (Ranke, 2015; Fujimoto et al., 2019; Baxter, 2024). For instance, the potential involvement of IGFBP-3 in endothelial dysfunction and smooth muscle cell proliferation in PAH warrants further exploration.

Emerging evidence suggests that IGFBP-3 may also interact with other signaling molecules implicated in vascular remodeling, such as transforming growth factor-beta (TGF-β) and vascular endothelial growth factor (VEGF), both of which play pivotal roles in PAH (Hudson et al., 2014). This hypothesis suggests a complex network of interactions that could provide insights into the mechanisms of PAH. However, there is a need for more targeted studies to validate these proposed pathways and better understand the contribution of IGFBP-3 to PAH progression. Future research should focus on investigating the molecular interactions of IGFBP-3 in the context of PAH, potentially uncovering novel therapeutic targets.

### 3.4 Insulin-like growth factor binding protein 4(IGFBP-4)

IGFBP-4 is a key IGFBP family member that regulates IGF-1 and IGF-2 bioavailability while also influencing cell proliferation, differentiation, and tissue remodeling through IGF-independent mechanisms. It primarily exerts inhibitory effects on cell proliferation and differentiation by sequestering IGFs and limiting their receptor-mediated signaling, although its role may vary depending on the cellular context and disease state (Yang et al., 2017; Lee et al., 2018; Walker et al., 2007). Notably, IGFBP-4 contributes to cardiovascular health by modulating vascular smooth muscle cells, maintaining endothelial function, and driving disease-related vascular remodeling (Peng et al., 2024).

As evidenced by the research conducted by Torres et al., IGFBP-4 has been implicated in the progression of PAH by driving vascular wall thickening through the promotion of pulmonary arterial vascular smooth muscle cell (VSMC) proliferation and the inhibition of apoptosis. This effect is mediated via the regulation of the IGF-1/IGF-1R signaling axis and the activation of downstream PI3K/Akt and MAPK/ERK pathways. Additionally, IGFBP-4 influences pulmonary artery endothelial cell function by inducing endothelial-mesenchymal transition (EndMT), contributing to vascular remodeling and dysfunction. Furthermore, it may amplify the inflammatory response by engaging the NF-κB pathway, further accelerating PAH progression (Torr et al., 2023). Given its critical involvement in these pathological mechanisms, IGFBP-4 represents both a potential biomarker for disease severity and a promising therapeutic target for PAH treatment.

### 3.5 Insulin-like growth factor binding protein 5 (IGFBP-5)

IGFBP-5 is a key IGFBP family member that regulates IGF-1 and IGF-2 activity and exerts IGF-independent functions on cells (Bach et al., 2005; Ding et al., 2016). Compared to IGFBP-4, IGFBP-5 plays a broader role in cell proliferation, differentiation, migration, and fibrosis. It promotes tissue repair in the muscular and skeletal systems, modulates vascular and fibroblast function in the cardiovascular system, and contributes to fibrosis in the lungs and kidneys (Zhu et al., 2024). Additionally, it regulates tumor progression by influencing the extracellular matrix (ECM) and signaling pathways (Lin W. et al., 2023). Its diverse functions make it a major focus in disease research and potential therapeutic development, especially in PAH.

Mechanistically, IGFBP-5 modulates ECM stability and the fibrinolytic system through direct interaction with plasminogen activator inhibitor-1 (PAI-1). Nam et al. further showed that this interaction depends on the basic heparin-binding domain of IGFBP-5 and can occur independently of IGF, enhancing tPA-mediated plasmin generation and subsequently regulating plasmin activity and MMP activation (Nam and Clemmons, 1997). Moreover, binding to ECM components or heparin-like glycosaminoglycans further modulates IGFBP-5–PAI-1 signaling, influencing its IGF-independent effects on ECM remodeling (Forbes et al., 2012; Sorrell et al., 2006). IGFBP-5 has been repeatedly associated with pro-fibrotic phenotypes in lung and other tissues: it induces ECM gene expression and fibroblast activation *in vitro* and promotes tissue fibrosis in multiple *in vivo* models (Yasuoka et al., 2009; Su et al., 2015). At the same time, some experimental contexts report attenuating or different effects on ECM turnover that appear to depend on cellular state, experimental conditions, or assay endpoints. These apparently conflicting findings likely reflect systematic differences across studies - for example, variation in animal models (hypoxia, monocrotaline, Sugen/hypoxia and transgenic overexpression models), differences in fibroblast phenotype (activated/myofibroblast versus quiescent fibroblast), and differences in measurement approaches (protein-level assays, activity assays versus mRNA expression). Comparative reviews of PAH models and translational limitations underscore that model choice and timing profoundly shape observed fibrotic and inflammatory phenotypes, and therefore can account for discrepant reports linking IGFBP-5 to fibrosis in pulmonary vascular disease (Voelkel and Bogaard, 2021; Maarman et al., 2013).

In summary, current evidence supports IGFBP-5 as a mechanistic regulator of ECM remodeling through IGF-independent interactions with the fibrinolytic system. However, its net effect on pulmonary fibrosis appears context-dependent. Clarifying these mechanisms will require standardized, cell-type–specific and temporally resolved *in vivo* studies that include functional activity endpoints such as plasmin and MMP activity. A clear understanding of IGFBP-5’s context-dependent actions will be essential for its rational targeting as a therapeutic strategy in PAH.

### 3.6 Insulin-like growth factor binding protein 6 (IGFBP-6)

IGFBP-6 is a protein that binds IGF-2 with high affinity, inhibiting its activity and regulating cell proliferation, differentiation, and migration (Sorrell et al., 2006). It plays a key role in growth, development, and the progression of diseases such as cancer, cardiovascular disease, and PH. Beyond its IGF-dependent functions, IGFBP-6 may also act through IGF-independent mechanisms. It shows anticancer potential by inhibiting cancer cell proliferation and migration (Oliva et al., 2018; Longhitano et al., 2022; Bach et al., 2013). In cardiovascular disease, alterations in IGFBP-6 levels can affect myocardial fibrosis, vascular function, and inflammation (Liu et al., 2020).

Recent studies suggest that IGFBP-6 might play a role in PAH, a condition characterized by vascular remodeling and smooth muscle cell proliferation in the pulmonary arteries. Although direct evidence linking IGFBP-6 to PAH is limited, its established roles in regulating vascular smooth muscle cell behavior and inflammation, as observed in other cardiovascular diseases, indicate it may influence the pathogenesis of PAH through similar mechanisms (Wang et al., 2020; Venuto et al., 2023; Wang et al., 2022). Elevated levels of IGFBP-6 in circulation and tissues have been observed in conditions such as atherosclerosis, suggesting that IGFBP-6 might contribute to the remodeling process in PAH (Shkurnikov et al., 2024; Su et al., 2025). However, further research is needed to validate these findings and clarify whether IGFBP-6 directly modulates pulmonary vascular changes in PAH.

Moreover, IGFBP-6 is known to exert its biological effects through both IGF-dependent and IGF-independent mechanisms, including interactions with IGF-2 and alternative signaling pathways such as JNK/ERK (Mohan and Baylink, 2002; Bach, 2015). While its role in PAH remains to be fully explored, its established effects on vascular smooth muscle cells and inflammation suggest potential relevance to PAH pathogenesis.

In conclusion, while the role of IGFBP-6 in PAH remains largely speculative, its known functions in cardiovascular disease and its potential to regulate fibrosis and inflammation suggest that IGFBP-6 may influence the development of PAH. However, much remains to be learned, and future studies will be crucial in confirming its involvement and potential as a therapeutic target or biomarker in PAH.

### 3.7 Insulin-like growth factor binding protein 7 (IGFBP-7)

IGFBP-7 is a multifunctional protein with a weak affinity for IGF, primarily exerting its effects through IGF-independent pathways. It plays a crucial role in regulating cell proliferation, apoptosis, vascular function, fibrosis, and immune responses (Fejzo et al., 2019; Chen et al., 2020; He et al., 2024). In cancer, its role is context-dependent, acting as a tumor suppressor in some cancers (e.g., melanoma) while promoting tumor progression in others (e.g., breast cancer) (Chien and Lowe, 2008; Chen et al., 2010; Akiel et al., 2017; Wilcox et al., 2021). In the cardiovascular system, IGFBP-7 has been linked to myocardial fibrosis, endothelial dysfunction, and PAH (Ko et al., 2022; Aboumsallem and de Boer, 2024; Katoh et al., 2024). Notably, it serves as a biomarker with potential diagnostic value in heart failure and acute kidney injury (Vaduganathan et al., 2022).

The TGF-β and bone morphogenetic protein (BMP) signaling pathways have been demonstrated to play a pivotal role in the pathogenesis of PAH, as previously evidenced by Juan C et al. (Chen et al., 2016). Moreover, evidence suggests that IGFBP-7 can modulate these pathways (Pen et al., 2008). Consequently, the potential for IGFBP-7 to regulate the progression of PAH by modulating specific pathways warrants further investigation. Moreover, recent multicenter cohort studies by Torres G et al. confirmed that serum IGFBP-7 levels are significantly elevated in PAH patients and independently associated with right heart dysfunction, reduced exercise capacity, and poor prognosis. Mechanistically, IGFBP-7 is highly expressed in pulmonary vascular endothelial and smooth muscle cells of PAH patients and may contribute to vascular remodeling by stimulating prostacyclin production. Notably, IGFBP-7 is markedly elevated in hepatopulmonary syndrome-associated PAH and strongly correlates with clinical outcomes. These findings highlight IGFBP-7 as a novel biomarker reflecting PAH progression and a potential therapeutic target via the prostacyclin pathway (Torres et al., 2023).

Table 2 summarizes the IGFBPs associated with the PAH.

**TABLE 2:** The role that IGFBPs play in biological functions, cardiovascular functions and PAH.

Biomarkers	References	Biological functions	Cardiovascular functions	Role in PAH and clinical implications
IGFBP-1	Slater et al. (2019)	It can regulate the bioavailability and activity of IGFs	IGFBP-1 influences smooth muscle cell proliferation and migration by binding to IGFs	It may contribute to PAH pathogenesis by modulating IGF bioavailability, influencing PASMC proliferation and migration, and potentially linking metabolic dysregulation to vascular remodeling. and It requires further validation before clinical translation
IGFBP-2	Byun et al. (2016) Nies et al. (2022) Yang et al. (2020)	IGFBP-2 plays a significant role in regulating cell proliferation, metabolism, and vascular remodeling	It may promote tumor progression and pathological vascular changes in cancer and cardiovascular disease, while exerting protective metabolic effects in conditions such as insulin resistance and obesity	IGFBP-2 contributes to PAH by stimulating PASMC proliferation via integrin/PI3K/Akt pathway and other non–IGF pathways, regulating PTEN, and linking vascular inflammation to remodeling; its elevated circulating and tissue levels correlate with disease severity and prognosis. and Its elevated circulating/tissue levels correlate with disease severity and prognosis, supporting its potential as a prognostic biomarker and therapeutic target.
IGFBP-3	Deng et al. (2018) Ding et al. (2023) Hudson et al. (2014)	IGFBP-3,the most abundant member of the IGFBP family, modulates cell proliferation and apoptosis via IGF-independent mechanisms and inhibits various malignant tumors	It contributes to vascular remodeling, endothelial function modulation, and the progression of atherosclerosis	It regulates IGF signaling and apoptosis via IGF-independent mechanisms, potentially influencing endothelial dysfunction and vascular remodeling in PAH. and Its role remains incompletely defined; future studies may clarify its biomarker and therapeutic relevance
IGFBP-4	Lee et al. (2018) Torr et al. (2023)	It primarily inhibits cell proliferation and differentiation by binding IGFs and blocking their receptor-mediated signaling, though its effects may vary with cellular context and disease state	IGFBP-4 modulates vascular smooth muscle cells, maintaining endothelial function, and driving disease-related vascular remodeling	IGFBP-4 can accelerates PAH progression by driving PASMC proliferation and EndMT through IGF-1/IGF-1R signaling, PI3K/Akt, and MAPK/ERK pathways, contributing to vascular wall thickening and inflammation. and It represents a candidate biomarker and potential target for IGFBP-4–directed inhibition
IGFBP-5	Zhu et al. (2024) Nam and Clemmons (1997) Maarman et al. (2013)	IGFBP-5 regulates cell proliferation, differentiation, migration, and fibrosis, and plays a promotive role in tissue repair, particularly within the muscular and skeletal systems	It can modulate vascular and fibroblast function in the cardiovascular system	IGFBP-5 is a context-dependent regulator of ECM remodeling via IGF-independent interaction with PAI-1 and modulation of fibrinolytic activity. It is generally pro-fibrotic in PAH models but may exert anti-apoptotic effects in specific contexts. and It represents a promising but complex therapeutic target.
IGFBP-6	Liu et al. (2020) Venuto et al. (2023) Mohan and Baylink (2002)	It plays a key role in growth, development, and the progression of diseases such as cancer, cardiovascular disease, and PAH.	The alterations in IGFBP-6 levels can affect myocardial fibrosis, vascular function, and inflammation	IGFBP-6 likely contributes to PAH through effects on vascular smooth muscle proliferation, inflammation, and fibrosis, though evidence is indirect. Elevated levels in atherosclerosis suggest potential relevance. and It may represent an early-stage biomarker/target pending further validation
IGFBP-7	Vaduganathan et al. (2022) Pen et al. (2008) Torres et al. (2023)	It plays a crucial role in regulating cell proliferation, apoptosis, vascular function, fibrosis, and immune responses, and exhibits a dual regulatory role in cancer	IGFBP-7 has been linked to myocardial fibrosis, endothelial dysfunction.And it serves as a biomarker with potential diagnostic value in heart failure and acute kidney injury	IGFBP-7 promotes vascular remodeling via modulation of TGF-β/BMP pathways and prostacyclin production. It is elevated in PAH (especially hepatopulmonary syndrome-associated PAH) and correlates with right heart dysfunction and poor prognosis. and It is a strong candidate biomarker and potential therapeutic target.^a^

^a^ PAH, pulmonary arterial hypertension; IGF, Insulin-like Growth Factor; IGFBP, Insulin-like Growth Factor Binding Protein; IGFBP-1, Insulin-like Growth Factor Binding Protein 1; IGFBP-2, Insulin-like Growth Factor Binding Protein 2; IGFBP-3, Insulin-like Growth Factor Binding Protein 3; IGFBP-4, Insulin-like Growth Factor Binding Protein 4; IGFBP-5, Insulin-like Growth Factor Binding Protein 5; IGFBP-6, Insulin-like Growth Factor Binding Protein 6; IGFBP-7, Insulin-like Growth Factor Binding Protein 7; PASMC, pulmonary arterial smooth muscle cell; PI3K, Phosphoinositide 3-kinase; Akt, Protein kinase B (PKB); PTEN, phosphatase and tensin homolog; EndMT, Endothelial-mesenchymal transition; MAPK, Mitogen-activated protein kinase; ERK, extracellular signal-regulated kinase; ECM, extracellular matrix; PAI-1, Plasminogen activator inhibitor-1; TGF-β, Transforming growth factor-beta; BMP, bone morphogenetic protein.

## 4 Insulin-like growth factor binding protein-likes (IGFBPLs)

The term IGFBPLs refers to a group of proteins that share structural similarities with the IGFBPs. This group currently includes IGFBPL1, IGFBPL2, and IGFBPL3. IGFBPL2, also known as IGFBP-7, has been previously discussed in the section on IGFBPs, while IGFBPL3 remains less studied, and its specific function is still unclear. Therefore, the following section will focus on exploring the potential relationship between IGFBPL-1 and PAH.

### 4.1 Insulin-like growth factor binding protein-like 1 (IGFBPL-1)

IGFBPL-1 shares sequence similarities with IGFBPs, suggesting a potential role in binding IGFs and modulating their activity. However, its precise biological function is not yet fully understood. It has been implicated in neurodevelopment, tumor suppression, and cell survival regulation, with high expression in the central nervous system (Butti et al., 2022; Jones and Clemmons, 1995). Some studies suggest it may influence cell proliferation and apoptosis, independent of IGF binding (Liu et al., 2013).

As previously discussed, IGFBPs play a role in vascular remodeling, smooth muscle cell proliferation, and endothelial function, all of which are critical processes in PAH pathogenesis. While IGFBPL-1’s specific involvement in PAH remains largely unexplored, its structural similarity to IGFBPs raises the possibility that it may influence vascular remodeling and inflammation, key drivers of PAH progression (Hwa et al., 1999; Pan et al., 2023). Further research is needed to clarify its potential as a biomarker or therapeutic target in PAH.

Table 3 outlines the IGFBPL-1 associated with the PAH.

**TABLE 3:** The role that IGFBPL-1 plays in biological functions, cardiovascular functions and PAH.

Biomarkers	References	Biological functions	Cardiovascular functions	Role in PAH
IGFBPL-1	Hwa et al. (1999)	IGFBPL-1 may influence cell proliferation and apoptosis, independent of IGF binding	Its structural similarity to IGFBPs raises the possibility that it may influence vascular remodeling	This still requires further research^a^

^a^ PAH, pulmonary arterial hypertension; IGFBP, Insulin-like Growth Factor Binding Protein; IGFBPL-1, Insulin-like Growth Factor Binding Protein-Like 1.

## 5 Pathogenic mechanisms of PAH: roles of the IGF family

**FIGURE 1:**
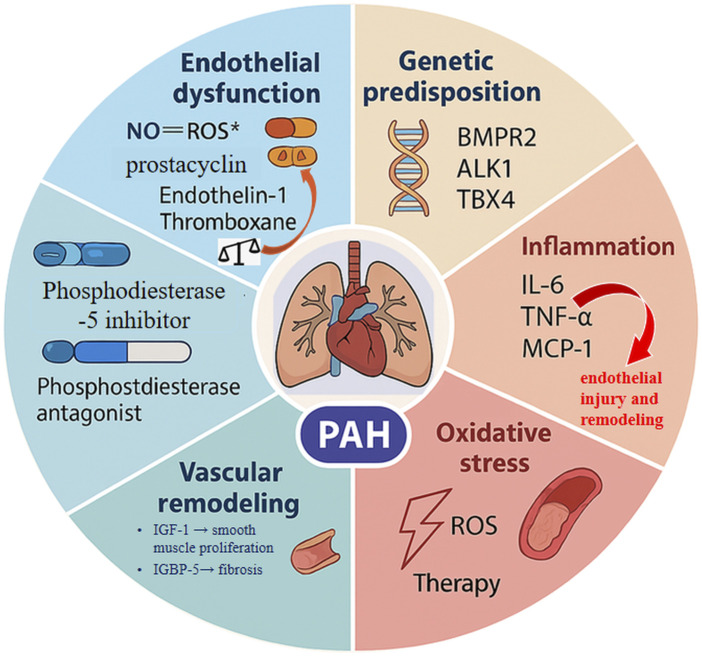
Proposed mechanisms involved in the development of PAH. PAH pathogenesis involves endothelial dysfunction, genetic predisposition, inflammation, oxidative stress, and vascular remodeling. Endothelial dysfunction results in an imbalance between vasodilators (e.g., nitric oxide, prostacyclin) and vasoconstrictors (e.g., endothelin-1), favoring vasoconstriction and vascular smooth muscle proliferation. Genetic mutations, such as in BMPR2 and ALK1, contribute to dysregulated vascular cell growth and resistance to apoptosis. Pro-inflammatory cytokines, including IL-6 and TNF-α, promote endothelial injury and remodeling. Oxidative stress, characterized by increased ROS and reduced antioxidant defenses, exacerbates vascular dysfunction and remodeling. Vascular remodeling includes smooth muscle cell hypertrophy and fibrosis, driven by mediators like PDGF and TGF-β. The IGF family intersects with these pathways, influencing vascular remodeling and fibrosis, and may serve as a potential therapeutic target in PAH.

PAH is a progressive disease characterized by elevated pulmonary vascular resistance, leading to right ventricular overload and heart failure. Multiple pathogenic mechanisms contribute to the development of PAH, including endothelial dysfunction, inflammation, oxidative stress, and vascular remodeling (Scherm et al., 2011; Leopold and Maron, 2016). These mechanisms interact to drive disease progression, as illustrated in Figure 1.

Endothelial dysfunction is a key early event in PAH, leading to an imbalance between vasodilators (e.g., NO, prostacyclin) and vasoconstrictors (e.g., endothelin-1, thromboxane) (Kurakula et al., 2021; Humbert et al., 2008). Reduced NO bioavailability and increased endothelin-1 levels promote vasoconstriction, thrombosis, and smooth muscle proliferation (Rizvi and Myers, 1997; Walsh et al., 2009). Therapies targeting endothelial dysfunction, such as phosphodiesterase-5 inhibitors and endothelin receptor antagonists, have shown efficacy in improving hemodynamics and symptoms in PAH patients (Frumkin, 2012; Madonna and Cocco, 2016; Channick et al., 2013). Recent evidence suggests that the IGF family is intricately involved in the pathogenesis of endothelial dysfunction in PAH. IGF-1 has been shown to exert protective effects on endothelial cells by activating the PI3K/Akt/eNOS signaling pathway, thereby enhancing NO production and promoting vasodilation (Muniyappa and Sowers, 2012; Isenovic et al., 2003). In contrast, IGF-1 may also contribute to vascular remodeling through the stimulation of PASMC proliferation and migration (Pluteanu and Cribbs, 2011). Notably, these proliferative effects of IGF-1 are potentiated by pro-inflammatory cytokines indicating a synergistic action with inflammatory signals that aggravates endothelial injury. Furthermore, several IGFBPs, particularly IGFBP-1, IGFBP-2, and IGFBP-3, are upregulated in PAH and may regulate IGF-1 bioavailability or exert IGF-independent actions that affect endothelial function and vascular homeostasis (Delafontaine et al., 2004). Importantly, these proliferative effects are potentiated by pro-inflammatory cytokines such as IL-6 and TNF-α, indicating that IGF-1 may act synergistically with inflammatory signals to aggravate endothelial injury and loss of barrier function.

Inflammatory cytokines such as interleukin-6 (IL-6), tumor necrosis factor-alpha (TNF-α), and monocyte chemoattractant protein-1 (MCP-1) are elevated in PAH, contributing to endothelial damage and vascular remodeling (Steiner et al., 2009; El Chami and Hassoun, 2012; Groth et al., 2014). Studies suggest immune cell infiltration in pulmonary arteries exacerbates disease progression (Hu et al., 2020). While anti-inflammatory therapies, including corticosteroids and immunosuppressants, have been explored, their long-term benefits remain uncertain (Sanchez et al., 2006). Interestingly, the IGF family, is increasingly implicated in modulating immune and inflammatory responses across various cardiovascular and pulmonary diseases. In the context of PAH, emerging evidence suggests that components of the IGF axis may influence cytokine expression, immune cell recruitment, and inflammatory signaling pathways. For instance,IGF-1 has been reported to enhance IL-6 and MCP-1 expression through NF-κB and PI3K/Akt activation in vascular tissues (Liu et al., 2001; Che et al., 2002), which can in turn recruit monocytes and perpetuate vascular inflammation. This feed-forward loop links IGF-1 signaling to worsening endothelial dysfunction. Certain IGFBPs, such as IGFBP-3, exert immunomodulatory effects that may either amplify or dampen inflammatory signaling depending on local context (Mohan and Baylink, 2002). These IGF–cytokine interactions represent a key cross-point between vascular inflammation and structural remodeling, helping to explain how immune dysregulation accelerates fibrotic change in PAH. Unraveling these mechanisms may offer new perspectives for immunomodulatory interventions targeting the IGF pathway in PAH.

Oxidative stress plays a critical role in the pathogenesis of PAH, primarily through its effects on endothelial dysfunction, vascular remodeling, and cellular metabolic reprogramming. It is driven by an imbalance between excessive reactive oxygen species (ROS) production and insufficient antioxidant defenses. Major sources of ROS in the pulmonary vasculature include mitochondria, NADPH oxidases (NOX enzymes), and uncoupled eNOS (Forstermann et al., 2017; Incalza et al., 2018). Elevated levels of oxidative stress biomarkers—such as 8-isoprostane, malondialdehyde (MDA), and oxidized glutathione—have been reported in the plasma and lung tissues of PAH patients, and these levels are positively correlated with disease severity and progression (Bowers et al., 2004; Xu et al., 2022). ROS not only promote endothelial cell apoptosis and impair NO bioavailability, but also stimulate smooth muscle cell proliferation and extracellular matrix deposition, exacerbating vascular remodeling. Therapeutic strategies targeting oxidative stress, including antioxidant vitamins (e.g., C and E), statins, and mitochondria-targeted antioxidants like MitoQ, have demonstrated promising results in preclinical PAH models. However, their clinical efficacy remains to be fully established, and large-scale trials are warranted (Kelso et al., 2002; Smith et al., 2008). There is growing evidence that components of the IGF family may intersect with redox signaling pathways in vascular disease. IGF-1 stimulates mitochondrial ROS production via PI3K/Akt and MAPK/ERK cascades (Ribeiro et al., 2014), which in turn can activate NF-κB and promote inflammatory gene expression, further aggravating endothelial dysfunction. Additionally, IGFBP-3 and IGFBP-5 regulate oxidative stress responses (Yoo et al., 2011); by influencing ROS levels, they indirectly control apoptosis, survival, and ECM turnover, thus linking oxidative stress to vascular remodeling.

Vascular remodeling involves endothelial proliferation, smooth muscle hypertrophy, and fibrosis, increasing pulmonary vascular resistance (Jeffery and Morrell, 2002; Tajsic and Morrell, 2011; Shimoda, 2020). Growth factors like PDGF and TGF-β drive smooth muscle proliferation and extracellular matrix deposition (Majack et al., 1990; Martin-Garrido et al., 2013). Imaging studies confirm that fibrosis severity correlates with disease progression (Goten et al., 2021). Pharmacological efforts to target fibrosis in PAH have included agents such as pirfenidone and nintedanib, which have demonstrated antifibrotic efficacy in idiopathic pulmonary fibrosis (IPF). However, their utility in PAH remains under investigation, and robust clinical evidence supporting their effectiveness is still lacking (Chianese et al., 2024; Collins and Raghu, 2019). The IGF family, is increasingly recognized as a key modulator in the vascular remodeling processes characteristic of PAH. IGF-1 can stimulate the proliferation and migration of vascular smooth muscle cells and fibroblasts, in part by activating the PI3K/Akt and MAPK/ERK pathways. These signaling cascades also intersect with profibrotic mediators such as TGF-β and PDGF, enhancing ECM deposition and cellular resistance to apoptosis (Huang et al., 2021; Vivar et al., 2012). Also,IGFBP-5 has been shown to accumulate in fibrotic pulmonary tissues and can promote fibrosis through both IGF-dependent and -independent pathways, including upregulation of collagen synthesis and modulation of matrix–cell interactions (Yasuoka et al., 2006; Nguyen et al., 2021). Thus, IGFBPs act as integrators of oxidative stress and matrix remodeling, determining whether the vascular wall undergoes adaptive repair or maladaptive fibrosis. In PAH models, altered expression of IGF-1 and IGFBPs has been observed in the pulmonary vasculature, suggesting that the IGF axis actively contributes to the fibrotic and proliferative vascular remodeling characteristic of the disease (Yasuoka et al., 2019; Bayes-Genis et al., 2000; Wang J. et al., 2012). These insights highlight the IGF signaling network as a potential therapeutic target, particularly for antifibrotic strategies. Targeted modulation of IGF-related pathways may offer new avenues for limiting vascular remodeling, reducing ECM accumulation, and improving clinical outcomes in PAH patients.

Together, these observations position the IGF axis as a central integrator of endothelial dysfunction, inflammation, oxidative stress, and vascular remodeling. Rather than acting through isolated pathways, IGF ligands and binding proteins orchestrate upstream and downstream signals that regulate cellular proliferation, apoptosis resistance, ECM turnover, and immune responses within the pulmonary vasculature. This mechanistic convergence highlights a pathogenic “hub” whose dysregulation may explain the transition from reversible vascular changes to fixed remodeling. Elucidating the spatiotemporal regulation of IGF signaling and its crosstalk with key profibrotic and inflammatory pathways may provide critical insights into disease heterogeneity and progression. Furthermore, the translational potential of IGF-targeted modulation—either as monotherapy or in combination with current vasodilatory or antifibrotic strategies—warrants rigorous investigation. Future studies should aim to clarify the context-specific roles of IGF family members and assess their viability as biomarkers or therapeutic targets, thereby advancing precision medicine approaches in PAH.

**FIGURE 2:**
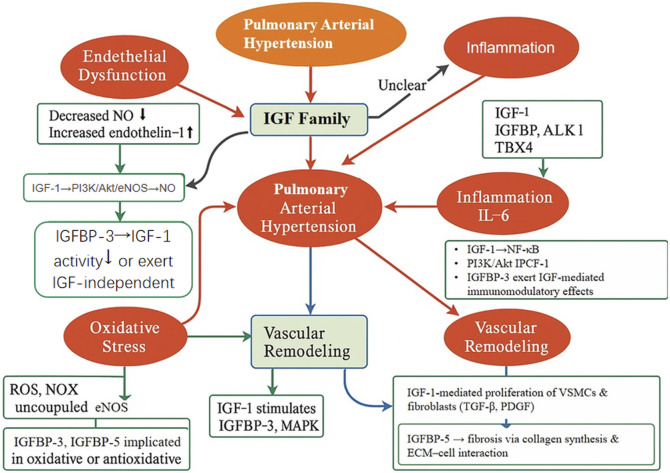
Schematic illustration of the proposed mechanisms through which the IGF family orchestrates key pathological processes in PAH. The IGF signaling axis is implicated in several major pathophysiological pathways contributing to PAH, including endothelial dysfunction, inflammation, oxidative stress, and vascular remodeling. IGF-1 enhances endothelial NO production through the PI3K/Akt/eNOS signaling cascade, while also promoting the proliferation of VSMCs and fibroblasts via the TGF-β and PDGF pathways. IGFBPs, particularly IGFBP-3 and IGFBP-5, exert both immunomodulatory and profibrotic effects, facilitating ECM accumulation and inflammatory responses. This figure integrates current mechanistic insights and underscores the IGF family’s potential as a central regulator and therapeutic target in PAH.

**FIGURE 3:**
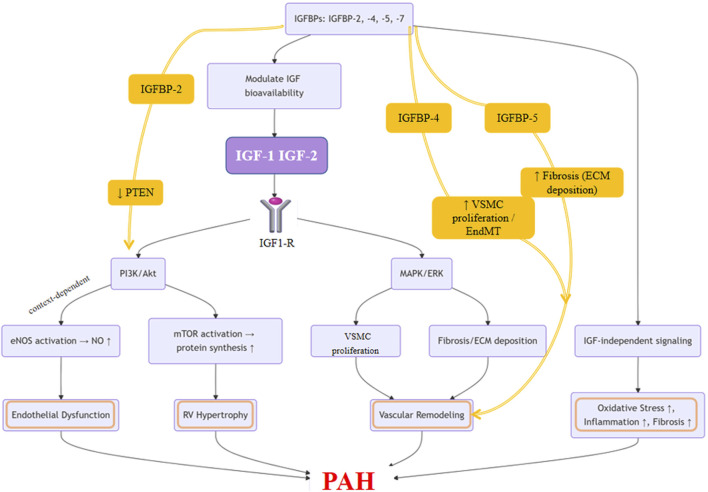
Integrated Mechanistic Model of IGF Family Signaling in Pulmonary Arterial Hypertension. This schematic illustrates the central role of the IGF axis as a pathogenic hub in PAH. The model highlights that binding of IGF-1 or IGF-2 to the IGF-1R activates downstream PI3K/Akt and MAPK/ERK signaling pathways. These pathways exert cell-type-specific effects: promoting PASMC proliferation, fibroblast activation, and ECM deposition, while in endothelial cells, contributing to NO production. The system is modulated by IGFBPs, with specific roles shown for IGFBP-2 (enhancing PI3K/Akt via PTEN inhibition), IGFBP-4 (promoting PASMC proliferation and endothelial-to-mesenchymal transition, EndMT), and IGFBP-5 (driving fibrosis). Crosstalk with other PAH pathways amplifies oxidative stress and inflammation, which together promote vascular remodeling, endothelial dysfunction, and pulmonary vascular resistance, ultimately culminating in disease progression.

As illustrated in Figure 2, the IGF family orchestrates multiple pathological processes involved in pulmonary vascular remodeling and the progression of PAH. Building upon this, Figure 3 presents an integrated mechanistic model highlighting how IGF signaling serves as a pathogenic hub that connects these processes through cell-type-specific effects, modulation by IGFBPs, and crosstalk with other PAH pathways.

## 6 Critical appraisal and unresolved questions

Although the preceding sections summarize the experimental and clinical literature linking the IGF axis to PAH, several unresolved contradictions remain in this field. These challenges arise not only from methodological heterogeneity but also from biological complexity, and they must be addressed to enable translation of mechanistic findings into clinical application.

First, most mechanistic data are derived from diverse preclinical models such as monocrotaline, chronic hypoxia, and genetically modified models, which differ in their predominant vascular lesions and time course of disease progression (Stenmark et al., 2009; Ryan et al., 2013). Moreover, variation in the timing of endpoint measurement across disease stages can result in seemingly contradictory findings, as illustrated by the dual role of IGF-1 signaling described earlier (Jin et al., 2014; Higashi et al., 2019).

Second, the cell-type specificity of IGF actions remains incompletely resolved. IGFs and IGFBPs exert distinct effects across multiple cell types: IGF-1 appears to exert protective effects in endothelial cells but pathogenic, pro-proliferative effects in PASMCs (Chetty et al., 2006). IGFs in adventitial fibroblasts have been associated with collagen deposition and fibrosis in experimental models, while IGFs in cardiomyocytes have been implicated in right ventricular hypertrophy and metabolic adaptation (Zhang and Lo, 1995; Wang KC. et al., 2012). These divergent effects likely reflect differences in receptor density, downstream signaling pathways, and local microenvironmental cues such as inflammation, hypoxia, and metabolic dysregulation. Moreover, most studies measure only circulating IGF or IGFBP levels, which provide useful clinical association data; however, they cannot distinguish whether the effects arise from local paracrine/autocrine signaling or from systemic endocrine exposure. Limited evidence in lung tissue and cell studies suggest that tissue‐level expression and local signaling may diverge from circulating levels in certain settings, underscoring this limitation (Yang et al., 2020; Schlueter et al., 2024).

Third, heterogeneity in measurement techniques and study design may account for some of the conflicting findings. Differences in assay platforms, antibody specificity, and data normalization strategies can yield variable IGF/IGFBP concentrations across studies. In addition, many clinical cohorts are relatively small, reducing statistical power. Uncontrolled confounders, including metabolic comorbidities, sex, age, and concomitant medications, may further obscure true associations.

Beyond these methodological and biological challenges, several core evidence gaps deserve emphasis. First, research on certain IGF family members—particularly IGF-2 and IGFBPLs—remains extremely limited, and most conclusions rely on indirect inference from structural homology or studies in unrelated disease contexts. Second, the cross-regulatory mechanisms linking IGF signaling with other key PAH pathways, such as BMP/TGF-β and HIF-1α, are poorly characterized, limiting our understanding of how these pathways converge to drive pulmonary vascular remodeling. Third, most available clinical data are observational (e.g., correlations between circulating IGFBP-2 levels and hemodynamic severity), whereas interventional evidence—such as studies testing IGF-1R inhibitors, IGF mimetics, or IGFBP modulators—is lacking.

Finally, many published studies are associative and cross-sectional, which limits causal inference. To address this gap, future studies should adopt cell-type-specific and temporally controlled genetic or pharmacologic interventions to clarify whether the IGF family plays predominantly protective or pathogenic roles under different contexts.

## 7 Clinical translation and future directions

The compelling association between the IGF axis and PAH pathogenesis unveils a promising frontier for therapeutic and diagnostic innovation. Translating this potential into clinical reality, however, demands a strategic and clear-eyed approach to overcome inherent challenges.

### 7.1 Therapeutic development

The compelling association between the IGF axis and PAH pathogenesis unveils a promising frontier for therapeutic and diagnostic innovation. Translating this potential into clinical reality, however, demands a strategic and clear-eyed approach to overcome inherent challenges.

Pulmonary Selectivity as a Central Challenge: The major obstacle in translating IGF-targeted therapies into PAH treatment is achieving pulmonary vascular selectivity while avoiding systemic metabolic and endocrine side effects.

Advanced Target Validation: The foremost challenge lies in achieving pulmonary vascular selectivity. Systemic inhibition of core components like IGF-1R risks significant off-target metabolic and endocrine side effects, as observed in oncology trials. Next-stage research must employ sophisticated *in vivo* models, such as cell-specific conditional knockout mice (e.g., targeting Igfbp4 in PASMCs or Igfbp7 in endothelial cells), to unequivocally establish causal roles and identify the most therapeutically tractable targets.

Potential Intervention Strategies: Several pharmacologic approaches could be explored to target the IGF axis in PAH. These include IGF-1R antagonists (e.g., linsitinib, teprotumumab) to suppress pathogenic IGF-1 signaling, IGFBP-4 or IGFBP-5 inhibitors to limit pro-fibrotic effects, and IGF mimetics or IGFBP-3 derivatives to restore protective IGF signaling in endothelial cells (Torr et al., 2023; Contois et al., 2012). RNA-based therapeutics (siRNA/ASOs) targeting overexpressed IGFBPs in PASMCs may offer tissue-selective modulation.

Repurposing and Novel Agents: A pragmatic strategy involves the rapid evaluation of existing IGF-1R inhibitors (e.g., linsitinib from oncology) in pre-clinical PAH models (Gulbins et al., 2023). Concurrently, efforts should focus on developing novel biologics or small molecules that achieve high selectivity for pathogenic IGFBP functions.

Biomarker-Enriched Trials: Future trials should stratify patients based on target expression (e.g., IGFBP-7 levels), combine IGF-axis interventions with standard therapies, and use advanced endpoints such as imaging markers of vascular remodeling.

### 7.2 Biomarker translation: from association to utility

The journey of circulating IGFBPs into clinical tools demands a rigorous, multi-stage process:

Analytical Validation: The initial step requires developing standardized, reproducible assays for candidates (e.g., IGFBP-2, -4, -7) that meet regulatory guidelines for precision, accuracy, and specificity.

Clinical Validation and Qualification: Large-scale prospective studies should demonstrate prognostic independence from established markers (e.g., NT-proBNP) and guide therapeutic decision-making.

Context-Specific Utility: Different IGFBPs may have distinct advantages in specific clinical scenarios. IGFBP-7 appears to be particularly specific for PAH associated with hepatopulmonary syndrome and may serve as a marker of vascular remodeling in this subgroup (Nies et al., 2022). IGFBP-2 has been correlated with disease severity and right ventricular dysfunction in pediatric PAH, supporting its use as a prognostic biomarker for risk stratification in children (Griffiths et al., 2020; Ploegstra and Berger, 2021). Future studies should compare the predictive value of individual IGFBPs and evaluate whether multi-marker panels improve diagnostic accuracy over single biomarkers.

Integration into Clinical Practice: Validated biomarkers could be deployed in multi-marker panels for refined risk stratification and as dynamic tools for monitoring treatment response and disease activity, ultimately enabling personalized management.

### 7.3 Overarching challenges and future directions

Future research priorities include clarifying the context-dependent duality of IGF-1, delineating its protective versus pathogenic roles across disease stages and cell types, and mapping crosstalk with core PAH pathways such as BMPR2/TGF-β signaling. Validation in human tissue will be essential, ideally through single-cell and spatial transcriptomic studies of PAH lung explants, to confirm cellular sources and signaling heterogeneity.

Moreover, the genetic underpinnings of IGF-axis involvement in PAH remain largely unexplored. Although genome-wide studies have identified pathogenic variants in canonical PAH genes such as BMPR2 and EIF2AK4, no reproducible associations have yet been reported for IGF ligands, receptors, or binding proteins. A few small-scale studies have linked IGF-axis polymorphisms to cardiometabolic traits (Vaessen et al., 2001; Horio et al., 2010), but these findings lack validation in PAH-specific cohorts. Future work integrating genome-wide association studies (GWAS), transcriptomic profiling, and functional assays will be critical to determine whether IGF-axis variants contribute to disease susceptibility or modify clinical trajectory.

In parallel, successful clinical translation will require rigorous evaluation of extra-cardiovascular safety profiles (e.g., metabolic and oncogenic risks) and careful assessment of optimal combination strategies with existing vasodilatory and antifibrotic therapies. Together, these efforts will refine our understanding of IGF biology in PAH and inform precision-medicine approaches for targeted intervention.

## 8 Conclusion

PAH remains a devastating disease with an urgent unmet need for therapies that directly target its progressive vascular pathology. This review synthesizes current evidence establishing the IGF family—including ligands, receptors, binding proteins (IGFBPs), and related proteins (IGFBPLs)—as a central regulatory hub in PAH pathogenesis. Beyond their classical metabolic roles, these molecules act as potent modulators of vascular remodeling, endothelial dysfunction, inflammation, and oxidative stress, intersecting with key pathways such as BMPR2 and TGF-β signaling.

The intricate, context-dependent actions of this axis—particularly the dual roles of IGF-1 and IGFBP-5—underscore the need for precision targeting strategies. Consistent upregulation of IGFBPs such as IGFBP-2, IGFBP-4, and IGFBP-7 in patient cohorts, together with their strong correlation with hemodynamic severity and survival, highlights their value as both mechanistic drivers and clinically actionable biomarkers.

Looking ahead, translating these insights into clinical practice offers a unique opportunity to shift PAH management from symptomatic relief toward true disease modification. Future research should prioritize cell-type-specific and temporally resolved studies to clarify protective versus pathogenic signaling, followed by rigorous analytical and clinical validation of candidate biomarkers. Ultimately, incorporating these biomarkers into patient stratification frameworks and designing biomarker-enriched, pulmonary-selective therapeutic trials could usher in an era of precision medicine, with the long-term goal of reversing pulmonary vascular pathology and improving survival.

## Abbreviations

PAH, Pulmonary arterial hypertension; IGF, Insulin-like growth factor; PH, Pulmonary hypertension; MAPK, Mitogen-activated protein kinase; NO, Nitric oxide; PI3K, Phosphoinositide 3-kinase; Akt, Protein kinase B (PKB); ERK, Extracellular signal-regulated kinase; PASMC, Pulmonary arterial smooth muscle cell; PTEN, Phosphatase and tensin homolog; PDGF, Platelet-derived growth factor; IGFBP, Insulin-like Growth Factor Binding Protein; TGF-β, Transforming growth factor-beta; TNF-α, Tumor necrosis factor-alpha; VEGF, Vascular endothelial growth factor; VSMC, Vascular smooth muscle cell; EndMT, Endothelial-mesenchymal transition; PAI-1, Plasminogen activator inhibitor-1; ECM, Extracellular matrix; MCP-1, Monocyte chemoattractant protein-1; BMP, Bone morphogenetic protein; IGFBPL, Insulin-like Growth Factor Binding Protein-Like; ROS, Reactive oxygen species; eNOS, Endothelial nitric oxide synthase; MDA, Malondialdehyde; IPF, Idiopathic pulmonary fibrosis; IL-6, Interleukin-6; GWAS, genome-wide association studies.
